# Cardiometabolic importance of 1-h plasma glucose in obese subjects

**DOI:** 10.1038/s41387-019-0084-y

**Published:** 2019-05-24

**Authors:** Lien Haverals, Kristof Van Dessel, An Verrijken, Eveline Dirinck, Frida Peiffer, Ann Verhaegen, Christophe De Block, Luc Van Gaal

**Affiliations:** Department of Endocrinology, Diabetology and Metabolism, Antwerp University Hospital, Wilrijkstraat 10, 2650 Edegem, Belgium

**Keywords:** Type 2 diabetes, Metabolic syndrome, Obesity

## Abstract

**Background/objectives:**

To study the importance and clinical usefulness of the 1-h plasma glucose (1hPG) in a Caucasian obese population with regard to the presence of prediabetes, diabetes, and metabolic syndrome (MetS).

**Subjects/methods:**

We conducted a cross-sectional study of 2439 overweight or obese subjects. All received an oral glucose tolerance test (OGTT) using the American Diabetes Association criteria. ROC-curves were used to compare the sensitivity and (1-specificity) of 1hPG versus FPG and 2hPG to diagnose prediabetes and diabetes.

**Results:**

Of 2439 patients (72.1% female) (age 43 ± 13 years, BMI 37.9 (34.6–41.6) kg/m^2^), 1262 (51.7%) had a 1hPG ≥ 155 mg/dL. The prevalence of prediabetes was 33.8% and of diabetes 9.8%. In these 240 diabetic patients, only 1.6% (four patients) did not show a 1hPG ≥ 155 mg/dL. Subjects with 1hPG ≥ 155 mg/dL were more insulin resistant (*p* < 0.001), had a higher waist (*p* < 0.001), visceral adipose tissue (VAT) (*p* < 0.001), systolic blood pressure (*p* < 0.001), microalbuminuria (*p* < 0.001), PAI-1 (*p* < 0.001), and worse lipid profile (*p* < 0.001) than subjects with 1hPG < 155 mg/dL. MetS was present in 64.1% of subjects with 1hPG ≥ 155 mg/dL versus 42.5% of subjects with 1hPG < 155 mg/dL (*p* < 0.001). In the group with 1hPG ≥ 155 mg/dL 32.6% had a normal glucose tolerance (NGT), 48.9% had prediabetes, and 18.5% was diagnosed with T2DM compared to 81.7% NGT, 17.7% prediabetes, and 0.6% T2DM in subjects with 1hPG < 155 mg/dL (*p* < 0.001). Among NGT subjects, 30.0% had a 1hPG ≥ 155 mg/dL and showed higher HOMA-IR (*p* = 0.008), VAT (*p* < 0.001), blood pressure (*p* < 0.001), and worse lipid profile (*p* = 0.001). Compared to 1hPG < 155 mg/dL, the sensitivity and specificity of 1hPG ≥ 155 mg/dL of prediabetes were 74.8% and 60.0% and for diabetes 97.1% and 53.2%, respectively.

**Conclusions:**

This study supports the role of 1hPG value as a valuable tool in the detection of obese subjects at high risk for T2DM and MetS.

## Introduction

Type 2-diabetes mellitus (T2DM) is increasingly prevalent and is associated with an increase in multimorbidity and mortality^1,2^. Therefore, screening and initiation of treatment are crucial^1,3^. For individuals at high risk of T2DM, including the obese population^4^, modifications in lifestyle, pharmacological interventions, and gastric bypass surgery can lower the incidence of T2DM and its complications^4–6^.

Traditionally, preventive counseling is launched after detecting prediabetes, defined as impaired fasting glucose (IFG) and/or impaired glucose tolerance (IGT). However, 40% of patients suffering from T2DM show normal glucose tolerance (NGT) at their first oral glucose tolerance test (OGTT)^1,3,7,8^. Following its worldwide standardization^2,9^, the HbA1c was accepted as a diagnostic test for diabetes in 2010 by the American Diabetes Association (ADA). HbA1c is a very stable parameter, convenient for patients and medical staff, but its cost and the influence of other medical conditions on its level, makes HbA1c less attractive as a screening tool.

In specific ethnic or geographic populations with NGT, a 1-h plasma glucose (1hPG) with a cut-off value of 155 mg/dL during OGTT was shown to be a valuable risk factor for the development of prediabetes and T2DM, respectively^1,3,9,10^. Indeed, 1hPG during OGTT might offer practical advantages over 2-h plasma glucose values (2hPG).

Moreover, cross-sectional studies indicated that subjects with increased 1hPG showed an increased risk for metabolic syndrome (MetS) and cardiovascular diseases^3,11,12^.

The aim of this study was to assess the value of a 1hPG in relation to the presence of prediabetes, diabetes, and MetS in a Caucasian obese population. Furthermore, we investigated the diagnostic sensitivity and practical use of the fasting plasma glucose (FPG) combined with the 1hPG, compared to the FPG combined with the 2hPG. Finally, we assessed the use of 1hPG among subjects with so called NGT as a suitable screening tool for MetS and cardiovascular risk factors.

## Materials (or subjects) and methods

### Participants

Patients visiting the obesity clinic at the Antwerp University Hospital for a problem of overweight or obesity were included. None of these patients were involved in a weight reduction program at the time of enrollment. Every patient underwent a standard metabolic work-up, approved by the Ethics Committee of the Antwerp University Hospital and provided written informed consent.

Inclusion of patients was based on age (≥18 years), completion of an OGTT and having a body mass index (BMI) ≥ 25 kg/m^2^. Patients with an established diagnosis of diabetes were excluded.

### Collection of data

A metabolic work-up, including a clinical examination with anthropometry, was performed in fasting conditions.

BMI was calculated as weight (measured with digital scale to 0.2 kg) over height (measured to 0.5 cm) squared. Waist circumference was measured between the lower rib margin and the iliac crest, while hip circumference was measured at the trochanter major’s level. Waist–hip ratio (WHR) was calculated dividing waist circumference by hip circumference. Bio-impedance analysis, as described by Lukaski et al.^13^, was used to determine body composition. Fat mass (FM%) was calculated using the formula of Deurenberg et al.^14^. Cross-sectional areas of total abdominal adipose tissue (TAT), visceral abdominal adipose tissue (VAT), and subcutaneous abdominal adipose tissue (SAT) were measured by computerized tomography (CT) at L4–L5 level according to previously described methods^15^.

Systolic and diastolic blood pressure was determined on the patient’s arm using a mercury sphygmomanometer after at least 5 min rest. A fasting blood analysis included lipid profile (total cholesterol, high-density lipoprotein-cholesterol (HDL-C), and triglycerides (TG)) and high-sensitive C-reactive protein (hs-CRP). Low-density lipoprotein-cholesterol (LDL-C) was calculated using the Friedewald formula^16^. An OGTT with 75 g glucose was performed, with glucose sampling at 0 (FPG), 15, 30, 60 (1hPG), 90, and 120 (2hPG) min and determination of insulin and C-peptide at 0, 30, 60, and 120 min. Insulin resistance was calculated, using the homeostasis model assessment (HOMA-IR) as described by Matthews et al.^17^, as (insulin (mU/L) * (glucose (mg/dL) * 0.0555)/22.5. HbA1c-data systematically have been collected since 2008.

Plasma glucose, total cholesterol, and TG were measured on Vitros 750 XRC (Ortho Clinical Diagnostics, Johnson & Johnson, UK). HDL-C was measured on Hitachi 912 (Roche Diagnostics, Germany). HbA1c was determined by high-performance liquid chromatography (Adams™ A1c HA- 8180, Arkray–Menarini instrument, Zaventem, Belgium; reference range: 4.8–6.0%). C-peptide was determined by electrochemiluminescence immunoassay on Modular E170 (Roche, Switzerland). Hs-CRP was assayed with nephelometry on BNII (Siemens Healthcare Diagnostics, Brussels, Belgium). Plasminogen activator inhibitor-1 (PAI-1) was determined with the Zymutest PAI-1 antigen kit (Hyphen BioMed, France) with a normal range of <5 ng/mL. Mean PAI-1 antigen levels in a group of 32 healthy volunteers ranged from 0.5 to 3.8 ng/mL.

### Classification of patients

Based on the criteria determined by the ADA, subjects were classified as NGT with FPG < 100 mg/dL associated with 2hPG < 140 mg/dL and HbA1c < 5.7%. Subjects were classified as IFG with FPG between 100 and 125 mg/dL, while subjects were classified as IGT with 2hPG between 140 mg/dL and 199 mg/dL, and/or HbA1c between 5.7% and 6.4%. Individuals with IFG and/or IGT were being referred to as having prediabetes^2^. Subjects referred to as probable diabetes were FPG ≥ 126 mg/dL and 2hPG ≥ 200 mg/dL or HbA1c ≥ 6.5%. Criteria for having diabetes were FPG ≥ 126 mg/dL and 2hPG ≥ 200 mg/dL and HbA1c ≥ 6.5%^2,18^.

MetS was defined based on the current harmonizing criteria^19–21^, based on the presence of any 3 out of the following 5 criteria^20^: Elevated waist circumference (with population- and country-specific definitions), elevated TG ≥ 150 mg/dL (or taking lipid lowering medication), reduced HDL-C < 40 mg/dL (male), or < 50 mg/dL (female), elevated blood pressure (systolic ≥ 130 mm Hg and/or diastolic ≥ 85 mm Hg) (or taking anti-hypertensive medication), and elevated FPG ≥ 100 mg/dL.

### Statistical analysis

All data were analyzed using statistical package for the social sciences (SPSS 21.0) software. Normality was checked using the Kolmogorov–Smirnov test. Variables that were not normally distributed were log transformed or square rooted when appropriate. Anthropometric measurements were presented as mean values with their standard deviation (SD) for normally distributed variables and median values with percentile 25 (P25) and percentile 75 (P75) for not normally distributed variables.

Categorical variables were tested with the chi-squared (*χ*^2^) test; differences in continuous variables were tested using independent sample Student’s *t* test (parametric variables) or Mann–Whitney *U* test (nonparametric variables). To test variables between more than two independent groups, one-way-Anova (parametric variables) or Kruskal–Wallis (nonparametric variables) was used when appropriate. To investigate which subgroups were significantly different from each other, a Tukey post hoc analysis, independent Student’s *t* test, or Mann–Whitney *U* test were performed as appropriate. Differences were considered significant at values of *p* < 0.05.

Linear regression with studentized residuals was used to test whether differences were independent of influencing factors. Results were considered significant if *p* < 0.05.

A multivariable linear stepwise regression analysis was used to examine parameters of the MetS associated with 1hPG. Collinearity diagnostics were performed to rule out independent association among variables.

Receiver operating characteristics (ROC) curves were used to compare the diagnostic sensitivity and (1-specificity) of 1hPG versus FPG and 2hPG to diagnose prediabetes and diabetes. ROC-curves were also used to calculate sensitivity and (1-specificity) for different cut-off values. Sensitivity, specificity, positive predictive value, negative predictive value, and accuracy for prediabetes and diabetes were calculated.

## Results

We included 2439 subjects, consisting of 680 (28%) men and 1759 (72%) women subjects, with a mean age of 43 ± 13 years and a median BMI of 37.9 (34.6–41.6) kg/m^2^. NGT was observed in 56.3% (*n* = 1374), while prediabetes was diagnosed in 33.8% (*n* = 825) and diabetes in 9.8% (*n* = 240). MetS was present in 1309 subjects (53.7%).

HbA1c values were available for 1138 patients. Based on HbA1c, 724 (63.3%) subjects showed normal HbA1c (<5.7%), 351 (30.8%) prediabetes (HbA1c 5.7–6.4%) and 63 (5.5%) diabetes (HbA1c ≥ 6.5%).

215 (8.8%) subjects took lipid lowering medication and 647 (26.5%) subjects took anti-hypertensive medication.

### Normal versus elevated 1hPG

In the studied cohort (*n* = 2439), 1262 (52%) subjects had a 1hPG ≥ 155 mg/dL. Based on OGTT, in the subgroup with 1hPG ≥ 155 mg/dL (elevated 1hPG group) 32.6% (*n* = 412) showed NGT, while 48.9% (*n* = 617) was diagnosed with prediabetes and 18.5% (*n* = 233) had T2DM. In comparison, in the subgroup with 1hPG < 155 mg/dL (normal 1hPG) 81.7% (*n* = 962) showed NGT, 17.6% (*n* = 208) had prediabetes and 0.6% (*n* = 7) had T2DM (*p* < 0.001) (Table 1). With regard to the aforementioned 9.8% de novo diagnosed diabetes patients, only 1.6% (four patients) did not show elevated 1hPG. Based on HbA1c, within the elevated 1hPG group 46.7% (*n* = 278) showed NGT, while 42.7% (*n* = 254) was diagnosed with prediabetes and 10.6% (*n* = 63) had T2DM. In comparison, within the normal 1hPG group 82.1% (*n* = 446) showed NGT, 17.9% (*n* = 97) had prediabetes and 0.0% (*n* = 0) had T2DM (*p* < 0.001).

**Table 1:** Metabolic parameters according to cut-off 155 mg/dL

	<155 mg/dL (*n* = 1 177)	≥155 mg/dL (*n* = 1 262)	*p*-value	Adjusted for age and gender
Age (year)	38.3 ± 12.1	47.2 ± 11.6	0.001	NA
Gender male/female (%)	22.3/77.7	33.1/66.9	<0.001	NA
Weight (kg)	103.4 (92.5–117.4)	106.8 (95.0–121.8)	0.001	<0.001
BMI (kg/m^2^)	37.6 (34.5–41.2)	38.3 (34.8–41.9)	<0.001	<0.001
Waist (cm)	111.7 ± 14.8	117.5 ± 14.6	<0.001	<0.001
WHR	0.91 (0.84–1.00)	0.99 (0.90–1.08)	<0.001	<0.001
Fat mass (FM%)	51.1 (42.6–61.4)	50.8 (42.3–60.8)	NS	0.044
TAT (cm^2^)	791.3 ± 177.0	844.2 ± 176.0	<0.001	<0.001
VAT (cm^2^)	161.8 ± 77.8	217.6 ± 92.3	<0.001	<0.001
SAT (cm^2^)	630.0 ± 150.5	625.8 ± 146.9	NS	NS
VAT/SAT	0.27 ± 0.18	0.37 ± 0.22	<0.001	0.026
Anti hypertensive drugs; yes (%)	188 (16.0)	459 (36.4)	<0.001	<0.001
Lipid lowering drugs; yes (%)	48 (4.1)	167 (13.2)	<0.001	<0.001
Bp syst (mm-Hg)	130 (119–140)	134 (122–143)	<0.001	<0.001
Bp diast (mm-Hg)	80 (70–83)	80 (73–87)	<0.001	<0.001
Total cholesterol (mg/dL)	203 (178–226)	209 (183–236)	<0.001	0.036
HDL-C (mg/dL)	50 (41–61)	48 (40–59)	0.002	0.003
LDL-C (mg/dL)	122.0 (101.0–145.1)	126 (104–150.4)	0.003	NS
Fasting TG (mg/dL)	124.0 (92.0–164.8)	149.0 (112.0–204.0)	<0.001	0.001
MetS yes/no (%)	42.5/57.5	64.1/35.9	<0.001	<0.001
HOMA-IR	2.56 (1.72–3.82)	3.64 (2.28–5.77)	<0.001	<0.001
hs-CRP (mg/dL)	0.53 (0.24–1.03)	0.52 (0.25–1.03)	NS	<0.001
Microalbuminuria (24 h)(µg/min)	6.5 (4.1–11.0)	7.0 (4.7–14.0)	<0.001	NS
PAI-1 (ng/mL)	1.4 (0.5–2.7)	2.2 (1.0–4.0)	<0.001	<0.001
Fibrinogen	393.2 ± 84.3	389.7 ± 85.5	NS	NS
Gluc 0 (mg/dL)	80 (75–85)	90 (83–99)	<0.001	<0.001
Gluc 60 (mg/dL)	130 (116–142)	185 (168–211)	<0.001	<0.001
Gluc 120 (mg/dL)	114 (97–133)	155 (129–185)	<0.001	<0.001
NGT/IFG/IGT/IFG+IGT/T2DM (%)	962/7/197/4/7	412/30/471/116/233		
	(81.7/0.6/16.7/0.3/0.6)	(32.6/2.4/37.3/9.2/18.5)	<0.001	<0.001
AUC glucose	20,130 (18,315–22,099)	26,497 (23,773–30,169)	<0.001	<0.001
HbA1c (%)	5.4 (5.2–5.6)	5.7 (5.4–6.0)	<0.001	<0.001
HbA1c: NGT/prediabetes/T2DM (%)	446/97/0	278/254/63	<0.001	<0.001
	(82.1/17.9/0.0)	(46.7/42.7/10.6)		
Fasting insulin (mlU/L)	13.4 (8.9–20.0)	16.4 (11.0–25.0)	<0.001	<0.001
Fasting C-peptide (nmol/L)	0.86 (0.64–1.10)	1.05 (0.77–1.40)	<0.001	<0.001

Data are expressed as mean ± SD or as median (Q1–Q3), if appropriate

*BMI* body mass index, *WHR* waist-hip ratio, *TAT* total abdominal adipose tissue, *VAT* visceral abdominal adipose tissue, *SAT* subcutaneous abdominal adipose tissue, *Bp diast* diastolic blood pressure, *Bp syst* systolic blood pressure, *HDL-C* high-density lipoprotein-cholesterol, *LDL-C* low-density lipoprotein-cholesterol, *TG* triglycerides, *HOMA-IR* homeostasis model assessment insulin resistance, *hs-CRP* high-sensitive C-reactive protein, *PAI-1* plasminogen activator inhibitor-1, *Gluc* plasma glucose, *AUC* area under the curve, *NGT* normal glucose tolerance, *IFG* impaired fasting glucose, *IGT* impaired glucose tolerance, *T2DM* type 2 diabetes mellitus, *HbA1c* hemoglobin A1c

In the elevated 1hPG group (*n* = 1262), 64.1% had the MetS versus 42.5% in the normal 1hPG group (*n* = 1177) (*p* < 0.001). Subjects in the elevated 1hPG group were older (*p* < 0.001), showed higher HbA1c (*p* < 0.001), higher 2hPG (*p* < 0.001), more insulin-resistance (HOMA-IR 3.64 versus 2.56, *p* < 0.001), had a higher waist (*p* < 0.001), higher VAT (218 ± 92 versus 162 ± 78 cm^2^, *p* < 0.001), higher systolic blood pressure (*p* < 0.001), higher total cholesterol (*p* < 0.001), higher TG (*p* < 0.001), lower HDL-cholesterol (*p* = 0.002), higher LDL-cholesterol (*p* = 0.003), higher microalbuminuria (*p* < 0.001), and had higher PAI-1 (2.2 (1.0–4.0) versus 1.4 (0.5–2.7) ng/mL, *p* < 0.001), compared with subjects with normal 1hPG. Given the significant difference in age and gender between those with normal and elevated 1hPG, analyses were repeated correcting for these two factors (see Table 1).

### Normal versus elevated 1hPG in subject with so called NGT

In a subgroup with NGT based on OGTT data (*n* = 1 374), 962 (70.0%) subjects showed normal 1hPG compared to 412 (30.0%) showing elevated 1hPG (Table 2). Those 412 subjects showed higher HOMA-IR (2.77 versus 2.50, *p* = 0.008), VAT (*p* < 0.001), blood pressure (*p* < 0.001), and a worse lipid profile (*p* = 0.001) than those with a normal 1hPG. Out of 412 subjects with elevated 1hPG, 191 (46.4%) had a MetS versus 381 out of 962 subjects (39.6%) with normal 1hPG (*p* < 0.001).

**Table 2:** Subjects with NGT: metabolic parameters according to cut-off 155 mg/dL

	<155 mg/dL (*n* = 962)	≥155 mg/dL (*n* = 412)	*p*-value	Adjusted for age and gender
Age (year)	37.7 ± 11.9	44.8 ± 11.7	<0.001	NA
Gender male/female (%)	20.7/79.3	32.0/68.0	<0.001	NA
Weight (kg)	102.0 (91.4–116.2)	104.6 (91.2–119.0)	NS	NS
BMI (kg/m^2^)	37.3 (34.3-41.0)	37.4 (34.3–41.2)	NS	NS
Waist (cm)	110.0 ± 14.7	114.4 ± 14.4	<0.001	0.051
WHR	0.90 (0.83–0.99)	0.96 (0.88–1.05)	<0.001	0.009
Fat mass (FM%)	50.5 (44.3–54.8)	48.1 (42.7–53.1)	0.001	NS
TAT (cm^2^)	736.8 ± 179.5	740 ± 180.6	NS	NS
VAT (cm^2^)	150.6 ± 73.2	185.5 ± 85.6	<0.001	NS
SAT (cm^2^)	586.1 ± 155.3	559.1 ± 151.8	0.004	NS
VAT/SAT	0.27 ± 0.19	0.34 ± 0.19	<0.001	NS
Anti hypertensive drugs; yes (%)	0 (0%)	0 (0%)	<0.001	<0.001
Lipid lowering drugs yes; (%)	0 (0%)	0 (0%)	<0.001	<0.001
Bp syst (mm Hg)	129 (119–140)	132 (120–140)	<0.001	0.045
Bp diast (mm-Hg)	80 (70–83)	80 (73–86)	<0.001	0.001
Total cholesterol (mg/dL)	203 (178–226)	207 (184–235)	0.001	0.079
HDL-C (mg/dL)	51 (42–61)	51 (42–62)	NS	NS
LDL-C (mg/dL)	122.0 (101.0–146)	123.0 (105.8–151.0)	0.033	NS
Fasting TG (mg/dL)	120.0 (90.0–160.2)	134.0 (99.3–188.0)	<0.001	0.022
MetS yes/no (%)	39.6/60.4	46.4/53.6	0.023	0.006
HOMA-IR	2.50 (1.65–3.73)	2.77 (1.84–4.30)	0.008	NS
hs-CRP (mg/dL)	0.52 (0.23–1.00)	0.50 (0.22–0.98)	NS	0.075
Microalbuminuria (24 h)(µg/min)	6.0 (3.6–10.1)	6.4 (4.1–11.1)	0.045	0.041
PAI-1 (ng/mL)	1.4 (0.5–2.9)	1.8 (0.8–3.0)	0.069	NS
Fibrinogen	391.33 ± 83.5	385.84 ± 88.5	0.307	NS
Gluc 0 (mg/dL)	80 (75–84)	86 (80–90)	<0.001	<0.001
Gluc 60 (mg/dL)	127 (113–138)	173 (162–186)	<0.001	<0.001
Gluc 120 (mg/dL)	108 (93–122)	118 (104–130)	<0.001	NS
AUC glucose	19,556 (17,968–21,017)	22,894 (21,474–24,251)	<0.001	0.091
HbA1c (%)	5.4 (5.2–5.5)	5.5 (5.3–5.7)	<0.001	0.005
HbA1c: NGT/prediabetes/T2DM: n (%)	348/62/0 (36.2/6.4/42.6)	116/59/1 (65.9/33.5/0.6)	<0.001	<0.001
Fasting insulin (mlU/L)	12.9 (8.6–19.0)	13.2 (9.4–20.4)	NS	0.065
Fasting C-peptide (nmol/L)	0.82 (0.61–1.05)	0.92 (0.66–1.18)	<0.001	0.042

Data are expressed as mean ± SD or as median (Q1-Q3), if appropriate

*BMI* body mass index, WHR waist-hip ratio, *TAT* total abdominal adipose tissue, *VAT* visceral abdominal adipose tissue, *SAT* subcutaneous abdominal adipose tissue, *Bp diast* diastolic blood pressure, *Bp syst* systolic blood pressure, *HDL-C* high-density lipoprotein-cholesterol, *LDL-C* low-density lipoprotein-cholesterol, *TG* triglycerides, *HOMA-IR* homeostasis model assessment insulin-resistance, *hs-CRP* high-sensitive C-reactive protein, *PAI-1* plasminogen activator inhibitor-1, *Gluc* plasma glucose, *AUC* area under the curve, *NGT* normal glucose tolerance, IFG impaired fasting glucose, *IGT* impaired glucose tolerance, *T2DM* type 2 diabetes mellitus, *HbA1c* hemoglobin A1c, *NA* not applicable

Based on HbA1c, in the elevated 1hPG group 65.9% (*n* = 116) showed NGT, while 33.5% (*n* = 59) was diagnosed with prediabetes and 0.6% (*n* = 1) had T2DM. In comparison, in the normal 1hPG 36.2% (*n* = 348) showed NGT, 6.4% (*n* = 62) had prediabetes and 42.6% had T2DM (*p* < 0.001).

Given the significant difference in age and gender between those with normal and elevated 1hPG in NGT subjects, analyses were repeated correcting for these two factors (Table 2).

### Metabolic parameters according to classification of diabetes

Based on available HbA1c data, subjects (*n* = 1063) was divided into four groups (NGT, IGT, probable diabetes, and confirmed diabetes). In the confirmed diabetes subgroup, subjects showed more weight (*p* < 0.001), had a higher BMI (*p* < 0.001), fasting TG (*p* < 0.001), microalbuminuria (*p* < 0.001), pai-1 (*p* < 0.001), higher prevalence in MetS (*p* < 0.001) Homa-IR (*p* < 0.001), AUC-glucose (*p* < 0.001), fasting glucose (*p* < 0.001), 1 h glucose (*p* < 0.001), 2 h glucose (*p* < 0.001), fasting insulin (*p* < 0.001), and fasting C-peptide (*p* < 0.001) in comparison with all other subgroups. Given the significant difference in age and gender between all classifications of diabetes, analyses were repeated correcting for these two factors (Table 3).

**Table 3:** Metabolic parameters according to classification of diabetes

	NGT (*n* = 464)	IGT (*n* = 549)	Probably diabetes (*n* = 88)	Diabetes (*n* = 22)	*p*-value	Adjusted for age and gender
Age (year)	37.64 ± 11.9	46.1 ± 12.4	51.0 ± 10.2	48.3 ± 13.3	<0.001^a,b,c,d^	NA
Gender male/female (%)	109/355	173/376	42/46	15/7.0	<0.001	NA
Weight (kg)	102.7 (91.2–117.4)	106.8 (96.3–124.3)	111.4 (100.2–122.8)	122.2 (112.5–140.0)	<0.001^a,b,c,d,e^	<0.001^a,c^
BMI (kg/m^2^)	36.9 (32.5–40.8)	38.0 (34.5–41.9)	38.7 (35.5–42.4)	40.7 (37.8–45.4)	<0.001^a,c^	<0.001^a,b,c^
Waist (cm)	111.5 ± 13.6	117.9 ± 14.0	122.5 ± 14.2	129.2 ± 10.1	<0.001^a,b,c,d,e^	<0.001^a,b,c^
WHR	0.90 (0.84–0.98)	0.98 (0.90–1.05)	1.05 (0.98–1.12)	1.08 (0.99–1.17)	<0.001^a,b,c,d,e^	<0.001^a,b,c,d^
Fat mass (FM%)	50.3 (44.3–54.5)	48.9 (42.7–54.3)	45.8 (39.4–53.5)	47.1 (42.0–53.9)	NS	NS
TAT (cm^2^)	753.1 ± 179.6	813.6 ± 179.6	809.0 ± 178.1	905.7 ± 186.3	<0.001^a,,c^	<0.001^a,c^
VAT (cm^2^)	157.0 ± 70	213.9 ± 88.8	265.9 ± 102.5	301.6 ± 101.0	<0.001^a,b,c,d,e^	<0.001^a,b,c,d,e^
SAT (cm^2^)	598 (483–713)	595 (491–703)	541 (450–633)	600 (475–684)	0.027^a,d^	0.001^a^
VAT/SAT	0.29 ± 0.17	0.39 ± 0.26	0.53 ± 0.28	0.53 ± 0.21	<0.001^a,b,c,d,e^	NS
Anti hypertensive drugs; yes (%)	79 (24.1)	197 (60.1)	41 (1.0)	11 (3.4)	<0.001^a,b,c,d,e^	<0.001^a,b,c,e,f^
Lipid lowering drugs; yes (%)	18 (14.8)	80 (65.6)	19 (15.6)	5 (4.1)	<0.001^a,b,c,e,f^	<0.001^a,b,c,e,f^
Bp syst (mm Hg)	75 (68–82)	78 (70–85)	78 (71–85)	80 (76–82)	<0.001^a,b,c^	0.024^a^
Bp diast (mm Hg)	123.5 (116–135)	129 (120–140)	131 (122–142)	134 (123–144)	0.002^a,b^	NS
Total cholesterol (mg/dL)	196 (174–221)	203 (177–229)	192 (164-233)	208 (178–236)	0.045^a^	NS
HDL-C (mg/dL)	50 (41–61)	48 (40–58)	44 (38–54)	40(34–52)	<0.001^a,b,c^	0.001^a,b,c,d^
LDL-C (mg/dL)	119 (98–142)	122 (102–145)	115 (87–148)	126 (90–143)	NS	NS
Fasting TG (mg/dL)	115 (83–160)	140 (108–187)	147 (101–202)	190 (132–309)	<0.001^a,b,c,e,f^	<0.001^a,b,c,e,f^
MetS yes (%)	162 (28.2)	320 (55.7)	71 (12.4)	21 (3.7)	<0.001^a,b,c,e,f^	<0.001^a,b,c,e,f^
HOMA-IR	2.41 (1.55–3.59)	3.40 (2.13–5.12)	5.47 (2.95–8.38)	12.05 (9.53–17.15)	<0.001^a,b,c,d,e,f^	<0.001^a,b,c,d,e,f^
hs-CRP (mg/dL)	0.46 (0.22–0.95)	0.61 (0.27–1.13)	0.34 (0.15–0.69)	0.74 (0.39–1.33)	NS	0.029^a,c,f^
Microalbuminuria (24 h)(µg/min)	6.2 (3.6–10.0)	7.0 (4.2–12.8)	9.0 (4.916.0)	16.8 (8.8–22.4)	<0.001^a,b,c,d,e,f^	NS
PAI-1 (ng/mL)	1.65 (0.80–3.23)	2.20 (1.00–3.80)	2.80 (1.50–5.30)	4.65 (3.55–9.30)	<0.001^a,b,c,d,e,f^	<0.001^a,b,c,d,e^
Fibrinogen	393.1 ± 82.7	402.2 ± 86.8	395.7 ± 79.9	420.9 ± 78.6	NS	NS
Gluc 0 (mg/dL)	80(76–86)	87 (80–95)	101 (94–111)	149 (130–191)	<0.001^a,b,c,d,e,f^	0.001^a,b,c,d,e,f^
Gluc 60 (mg/dL)	134 (117–155)	165 (146–189)	218 (197-241)	276 (245–312)	<0.001^a,b,c,d,e,f^	0.001^a,b,c,d,e,f^
Gluc 120 (mg/dL)	114 (97–127)	152 (139–168)	220 (209–243)	278 (271–338)	<0.001^a,b,c,d,e,f^	0.001^a,b,c,d,e,f^
AUC glucose	20,633 (18,833–22,331)	25,290 (23,321–27,608)	34,560 (32,381–37,920)	46,110 (41,838–53,128)	<0.001^a,b,c,d,e,f^	<0.001^a,b,c,d,e,f^
HbA1c (%)	5.3 (5.2–5.5)	5.7 (5.4–5.9)	6.2 (5.8–6.7)	7.9 (6.9–8.2)	<0.001^a,b,c,d,e,f^	0.001^a,b,c,d,e,f^
Fasting insulin (mlU/L)	12.1 (7.8–18.2)	15.5 (10.3–23.1)	19.2 (12.0–31.4)	33.4 (23.2–42.8)	<0.001^a,b,c,d,e,f^	0.001^a,b,c,d,e,f^
Fasting C-peptide (nmol/L)	0.90 (0.72–1.11)	1.11 (0.89–1.38)	1.46 (1.04–1.77)	1.77 (1.51–2.13)	<0.001^a,b,c,d,e,f^	0.001^a,b,c,d,e,f^

Data are expressed as mean ± SD or as median (Q1–Q3), if appropriate

*BMI* body mass index, *WHR* waist-hip ratio, *TAT* total abdominal adipose tissue, *VAT* visceral abdominal adipose tissue, *SAT* subcutaneous abdominal adipose tissue, *Bp diast* diastolic blood pressure, *Bp syst* systolic blood pressure, *HDL-C* high-density lipoprotein-cholesterol, *LDL-C* low-density lipoprotein-cholesterol *TG* triglycerides, *HOMA-IR* homeostasis model assessment insulin-resistance, *hs-CRP* high-sensitive C-reactive protein, *PAI-1* plasminogen activator inhibitor-1, *Gluc* plasma glucose, *AUC* area under the curve, *NGT* normal glucose tolerance, *IFG* impaired fasting glucose, *IGT* impaired glucose tolerance, *T2DM* type 2 diabetes mellitus, *HbA1c* hemoglobin A1c

Tuckey post hoc test/Mann–Whitney *U* test

^a^Comparing NGT to IGT (*p* < 0.05)

^b^Comparing NGT to probably diabetes (*p* < 0.05)

^c^Comparing NGT to diabetes(*p* < 0.05)

^d^Comparing IGT to probably diabetes (*p* < 0.05)

^e^Comparing IGT to diabetes(*p* < 0.05)

^f^Comparing probably diabetes to diabetes (*p* < 0.05)

### Elevated 1hPG versus MetS

In obese subjects, independent metabolic risk factors for a 1hPG ≥ 155 mg/dL were TG (odds ratio (OR) = 1.70; confidence interval (CI) = 1.42–2.04), FPG (OR = 20.98; CI = 12.37–35.58) and blood pressure (OR = 1.81; CI = 1.50–2.18), whereas HDL-C showed a trend in significance (OR = 0.83; CI = 0.71–1.02). There seems to be a difference in independent risk factors between men and women, where women with a higher TG value have 1.84 (1.48–2.28) times more chance to have a 1hPG ≥ 155 mg/dL and men with a lower HDL-C have 1.57 (1.10–2.25) times more chance of having a 1hPG ≥ 155 mg/dL.

When selecting NGT individuals, for subjects with a 1hPG ≥ 155 mg/dL, independent risk factors were TG (OR = 1.59; CI = 1.24–2.03), HDL-C (OR = 1.33; CI = 1.04–1.71), and blood pressure (OR = 1.61; CI = 1.25–2.07).

### Discriminative ability

**Fig. 1:**
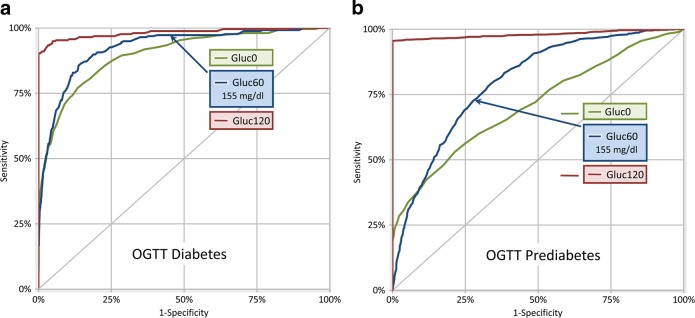
Receiver operating characteristic curve for he discriminatory ability of 1hPG values with respect to diabetes and prediabetes, based on OGTT diabetes criteria. **a** The ROC curve for FPG, 1hPG, and 2hPG to discriminate individuals with diabetes. **b** The ROC curve for FPG, 1hPG, and 2hPG to discriminate individuals with prediabetes, after excluding T2DM. The AUC for 2hPG (prediabetes, 0.837; diabetes, 0.982) was greater compared to 1hPG ≥ 155 mg/dL (prediabetes, 0.700; diabetes, 0.924) and FPG (prediabetes, 0.629; diabetes, 0.898)

Figure 1 represents ROC-curves constructed analyzing FPG, 1hPG-, and 2hPG-values to detect diabetes (Fig. 1a) and prediabetes (Fig. 1b) based on the OGTT criteria. The area under the curve (AUC) for 2hPG (prediabetes, 0.837; diabetes 0.982) was greater compared to 1hPG ≥ 155 mg/dL (prediabetes, 0.700; diabetes 0.924) and FPG (prediabetes 0.629; diabetes 0.898). The sensitivity and specificity of 1hPG ≥ 155 mg/dL with respect to prediabetes were 74.8% and 60.0%; and for diabetes 97.1% and 53.2%, respectively.

**Fig. 2:**
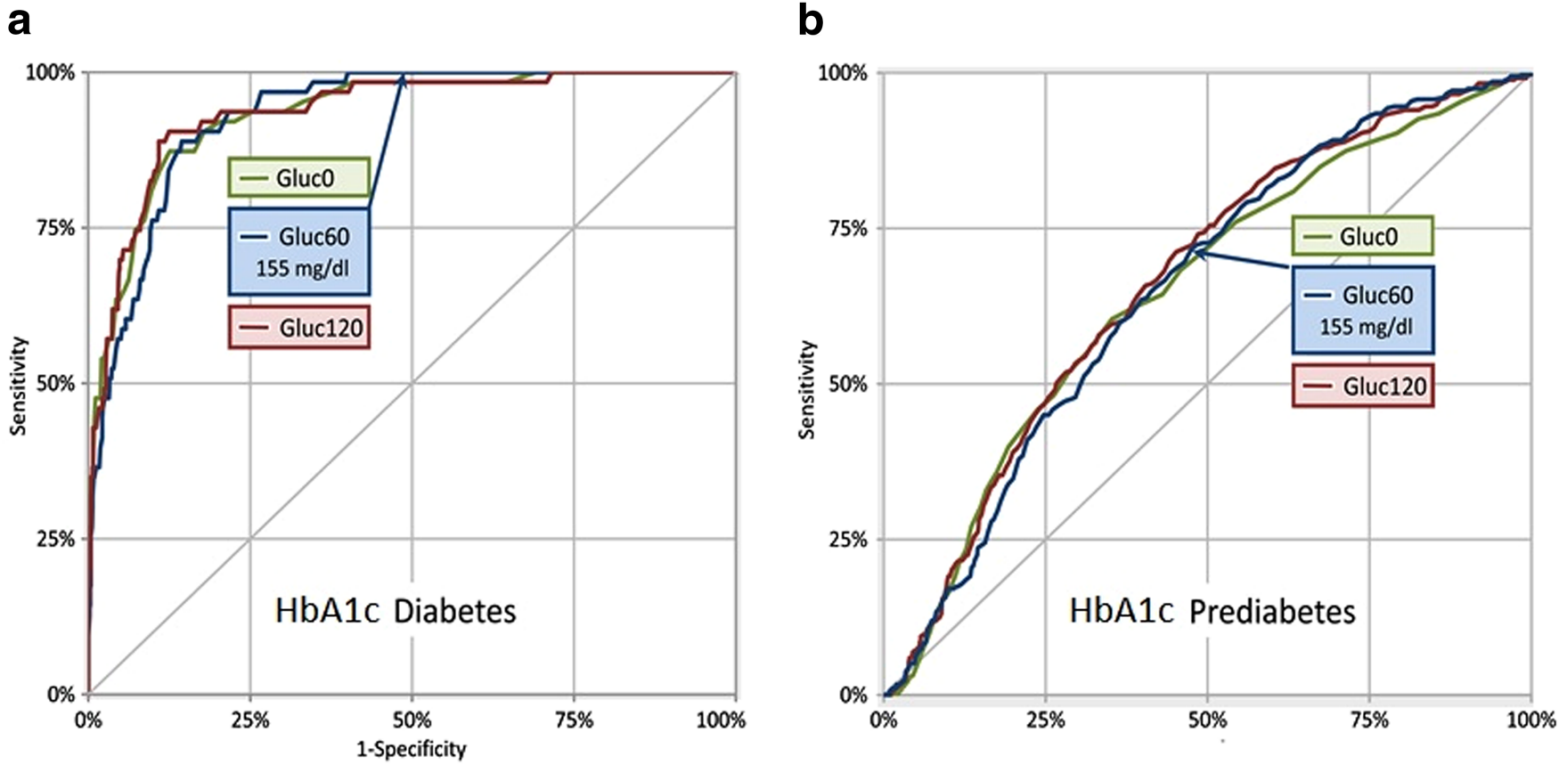
Receiver operating characteristic curve for the discriminatory ability of 1hPG values with respect to diabetes and prediabetes, based on HbA1c-levels. **a** The ROC curve for FPG, 1hPG, and 2hPG to discriminate individuals with diabetes. **b** The ROC curve for FPG, 1hPG, and 2hPG to discriminate individuals with prediabetes, after excluding T2DM. For diabetes (**a**), the AUC for FPG (0.935) was greater compared to 1hPG ≥ 155 mg/dL (0.933) and 2hPG (0.656). For prediabetes (**b**), the AUC for 2hPG (0.937) was greater followed by 1hPG ≥ 155 mg/dL (0.688) and FPG (0.669)

Figure 2 shows ROC-curves constructed analyzing FPG, 1hPG-, and 2hPG-values and diagnosis of diabetes and prediabetes based on HbA1c-level. For diabetes (Fig. 2a), the AUC for FPG (0.935) was greater compared to 1hPG ≥ 155 mg/dL (0.933) and 2hPG (0.656). For prediabetes (Fig. 2b), the AUC for 2hPG (0.937) was greater followed by 1hPG ≥ 155 mg/dL (0.688) and FPG (0.669). The sensitivity of 1hPG ≥ 155 mg/dL with respect to prediabetes was 72.4% and 56.7%; with respect to diabetes sensitivity and specificity, respectively, were 100.0% and 50.5%. All patients with de novo diagnosed diabetes based on HbA1c criteria showed an elevated 1hPG, where 27.0% (18 patients) did not show an elevated 2hPG.

## Discussion

This cross-sectional study reports on an obese Caucasian population, having undergone an OGTT during a metabolic workup, and stratified subjects based on ADA criteria. The 1hPG is rarely estimated or taken into account. The discrepancy about the 1hPG literature led to a search for its added value in screening for diabetes^22^.To the best of our knowledge, we are the first to study the added value of the 1hPG value in an obese population.

In specific ethnic and geographic populations, studies demonstrated the value of 1hPG with a cut-off value of 155 mg/dL during OGTT as a valuable predictor for progression to prediabetes and T2DM, respectively^1,3,9,10^. In a study by Priya et al.^3^, non-obese subjects with 1hPG ≥ 155 mg/dL were diagnosed with diabetes after a follow up of at least a year, while Fiorentino et al.^10^, made this conclusion in a five year follow up study^10^ and Abdul Ghani et al.^1^ after at least 7-year follow up^1^. This is a timeframe in which development of T2DM can be postponed or avoided through lifestyle intervention.

Our ROCs revealed that for identifying the prevalence of diabetes, a 1hPG ≥ 155 mg/dL during a patient's OGTT, is a useful cut-off point. Furthermore, elevated 1hPG among obese subjects is associated with an ~3 times higher incident detection of prediabetes, 30 times higher incident detection of T2DM and a 50% higher prevalence of MetS, compared to the group with normal 1hPG. These findings are in line with the study of Pareek et al.^8^, stating that subjects with 1hPG ≥ 155 mg/dL have a significantly increased risk of incident T2DM. Other studies, performed in specific settings with regard to ethnicity and geography, have also demonstrated the importance of 1hPG during an OGTT, suggesting that elevated 1hPG could facilitate clinicians in earlier detection of adults at risk for MetS, cardiovascular disease, and future T2DM^3,9,20,23,24^. Further analyses of metabolic risk factors in our study confirmed that they were independently associated with a 1hPG ≥ 155 mg/dL.

Moreover, International Diabetes Federation highlighted other parameters, appearing to be linked with MetS, which can be considered as additional criteria in predicting T2DM and/or cardiovascular disease. Those parameters are insulin resistance (HOMA-IR), microalbuminuria and “prothrombotic state” (to be measured via fibrinolytic factors such as PAI-1)^19,25,26^. Moreover, Succurro et al.^27^ added that NGT subjects with 1hPG > 155 mg/dL have an atherogenic profile similar to IGT subjects and supports that 1hPG > 155 mg/dL may be considered to identify individuals at risk for cardiovascular disease^27^. Screening and prevention of T2DM can be helped by assessment of IR, although cut-off values also are specific depending on race, age, gender, etc. PAI-1 is considered to be an important indicator of cardiovascular risk and strongly related to MetS, while microalbuminuria is used as an important marker for detection of renal dysfunction. Our study shows that there is a significant difference between subjects with a normal and an elevated 1hPG, with respect to cardiometabolic profile. When we focus on subjects with NGT, elevated 1hPG levels were associated with elevated HOMA-IR, presence of microalbuminuria and a worse lipid profile and a significant trend was found for PAI-1. Our results confirm those of the study of Abdul Ghani et al.^9^, who observed that the prevalence of MetS in people with NGT was 14.3%. In our study, we noticed a 15% higher prevalence of MetS in subjects with NGT, but an elevated 1hPG compared to subjects with a NGT and a normal 1hPG. However, published studies were population based, while we selected patients from an obesity clinic with a higher mean BMI. It is noteworthy that 1hPG is a convenient measure in an obese population to detect subjects at risk for MetS and cardiovascular disease, as it does not necessitate 2-h values^1,3,8–10,12^. Indeed, this study shows that interpreting an OGTT without taking into account 2hPG value only 1.6% (four patients) of diabetes diagnosis, based on OGTT criteria, would have been missed. On the other hand, all patients diagnosed with diabetes based on HbA1c values, showed an elevated 1hPG, while 18 patients did not show an elevated 2hPG. Therefore a shorter 75 g OGTT with FPG and 1hPG estimation can reduce the workload among nurses without significant risk of misdiagnosis. This seems to be in line with Fiorentino et al.^28^ who stated that a 1hPG > 155 mg/dL may be a useful tool to identify a subset of individuals within HbA1c-defined glycemic categories at higher risk of developing type 2 diabetes.

A major strength of this study is that it consists of 2439 well-characterized subjects, and this can be considered a large cohort. There was no significant influence from other medical conditions. A relative limitation is the small number of men analyzed in this study. The subjects included in this study were referred by their general practitioner or came at their own initiative and it is well known that women tend to seek help for weight problems more often and earlier than men. However, this finding does not alter the importance and conclusions of the observations. A final limitation is the cross-sectional study nature of this study. It would be interesting to organize a long-term follow-up to improve the prediction model.

## Conclusion

This study illustrates the clinical importance of a 1-h glucose determination in obese subjects. ROC analysis confirmed 155 mg/dL as a useful cut point to diagnose prediabetes or diabetes in obese patients. Moreover, even in subjects with a normal fasting glycemia, an elevated 1hPG can discriminate subjects having a worse cardiometabolic risk profile. As such, this group can be considered as a new target group for early intervention.
